# Research on Optimization of Height Difference of a Precision Horizontal Machining Center

**DOI:** 10.3390/mi15101279

**Published:** 2024-10-21

**Authors:** Lin Han, Zhenyun Zhang, Xueguang Tian, Houjun Qi, Fucong Liu, Yang Qi

**Affiliations:** 1 School of Mechanical Engineering, Tianjin University of Technology and Education, Tianjin 300222, China; 2 Tianjin High-End Intelligent CNC Machine Tool Engineering Research Center, Tianjin 300222, China

**Keywords:** machine tools, height difference of guideways, rigid–flexible system, static deflection, frequency response

## Abstract

This work proposes a methodology to determine the height difference of the guideways of machine tools where two guideways are not placed on the same horizontal plane. Firstly, a rigid–flexible coupling system consisting of a moving rigid mass and an elastic overhanging beam is presented as an equivalent mechanical model of a column and a spindle box. Then, the relationship between the deviation of a reference point and the height difference or the spindle box’s stroke is modeled. Next, the natural frequency and mode shape function of the overhanging beam, and the frequency response functions of the coupling system, are derived. The results indicate that there always exists an optimal height difference minimizing the relative deflection over the stroke of the moving part, and the optimal value depends on the loads in two directions and the stroke of the moving part. Similarly, there is also an optimal choice maximizing the first-order resonant frequency of the coupling system; however, the optimal solutions for both static and dynamic cases are not the same. This work provides beneficial instruction for choosing the height difference of machine tools with two guideways on a bed that are not on the same plane.

## 1. Introduction

Machine tools play a significant role in manufacturing. Structural design optimization is a crucial approach to improving the performance of machine tools. In today’s economic environment, the machine tool industry continually focuses on enhancing performance while reducing costs, conserving energy, and minimizing environmental impacts [1]. A lot of the work focuses on structural optimization or the lightweight design of components or a holistic machine tool. Shen et al. [2] adopted an adaptive growth method to design an inner stiffener layout of structures, like the headstock, column, and bed, and an optimization strategy for a holistic machine tool utilizing dynamic sensitivity was presented. Ma et al. [3] addressed a dynamic modeling and design methodology for a box-in-box-type precision horizontal machine tool based on parallel artificial neural networks and genetic algorithms, and lower-order natural frequencies and frequency responses over the task workspace were improved. Chan et al. [4] focused on the implementation of finite element methods for the analysis and optimization of CNC (Computerized Numerical Control) machine tool operations and the structure, and an increase of 1.5% in the first three modal frequencies was observed. Liu et al. [5] presented four types of bionic tables and compared them through the finite element method; then, a multi-objective optimization design for the prairie rushes bionic structure table was conducted. The results indicate that the natural frequency, mass, deformation, and maximum equivalent stress were all improved compared with the original design. Similarly, Chen et al. [6] employed finite element analysis to find key parts of a five-axis tool grinding machine. After model parameterization, a back propagation neural network and genetic algorithm were combined to solve a multi-objective optimization problem with the constraints of the first-order natural frequency, deformation, and mass. Xiao et al. [7] proposed a lightweight design method for machine tools based on particle damping technology and the finite element method. The results indicate that the acceleration and displacement amplitudes of a lightweight machine tool with a particle damper were smaller than those of traditional machine tools. Li et al. [8] conducted an optimization effort on the crossbeam of a gantry machine tool. The X-type structure of the internal unit of the crossbeam was replaced by an O-type one, and specific dimensions were optimized using a neural network algorithm and a nondominated sorting genetic algorithm, considering constraints of deformation, modal frequency, and mass. Triebe et al. [9] explored the lightweighting of the machine slides (or tables) to achieve energy savings, considering the table and workpiece mass, the cutting force, and the motor that drives the table. The results show potential energy savings of up to 38% in terms of the energy required to move the table. Ji et al. [10] investigated the structural optimization of the tool slide of a dry-cut hobbing machine, comprehensively considering energy consumption and the static and dynamic performance of the machine tool. Several methods like the uniform design method, sensitivity analysis, response surface method, principal component analysis, and hybrid algorithm combining particle swarm optimization with the simulated annealing algorithm were integrated in the work. Cui et al. [11] carried out the topological optimization of the cardan frame of an A/C swing angle milling head using the finite element method. Xie et al. [12] presented an optimization method based on the Kriging and multi-objective genetic algorithm to reduce the mass of a hinged beam structure of the cubic diamond press, taking the stress and displacement peak as the constraint conditions.

**Figure 1:**
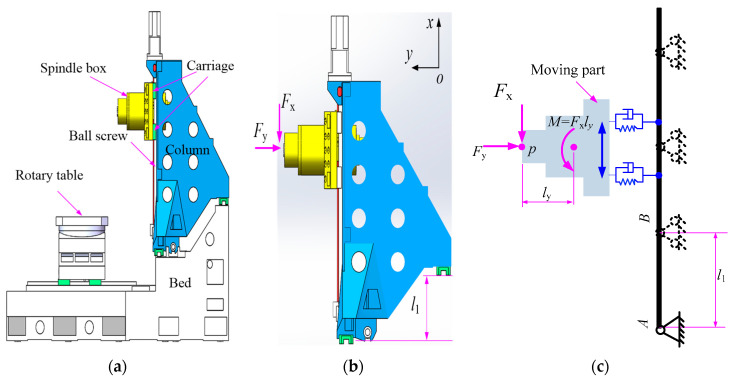
A horizontal machining center and simplified mechanics model. (**a**) Schematic of a horizontal machining center; (**b**) Research object of this work; (**c**) Simplified mechanics of the objective.

This work focuses on an innovative design of the machine tool structure, as Figure 1a shows. To improve the load-bearing capacity, reduce the mass of moving parts, and promote the machining efficiency of a horizontal machining center, two guideways connecting the column and bed are no longer placed on the same horizontal plane and there exists a height difference denoted by *l*_1_. However, the influence of the height difference on the end performance of the machine tool is not revealed yet, which is the motivation of this work.

Obviously, the parameter *l*_1_ mainly affects the static and dynamic behaviors in the *xy* plane; thus, only forces *F*_x_ and *F*_y_ are taken into account during the following analysis, as shown in Figure 1b. Transforming *F*_x_ into the centroid of the moving part, an equivalent moment *M* = *F*_x_*l*_y_ is obtained. For the further transformation of *F*_x_ to the axis of the ball screw, a moment and a force are applied to the ball screw–nut pair, which are neglected in the following analysis. Thus, the loads applied to the spindle box include *F*_y_ and moment *M*. As the inner structure of the column is hollow, and the research objective is to explore the influencing trend of the height difference, the column is simplified into an overhanging beam and the spindle box into a rigid body, and the two bodies are connected by the spring-damping unit, as shown in Figure 1c.

The following sections of this paper are organized as follows: The static deflection analysis is conducted in Section 2 and the frequency response functions of the coupling system are derived in Section 3. The simulation and discussions are presented in Section 4, and the conclusion is presented in the final section.

## 2. Static Analysis

**Figure 2:**
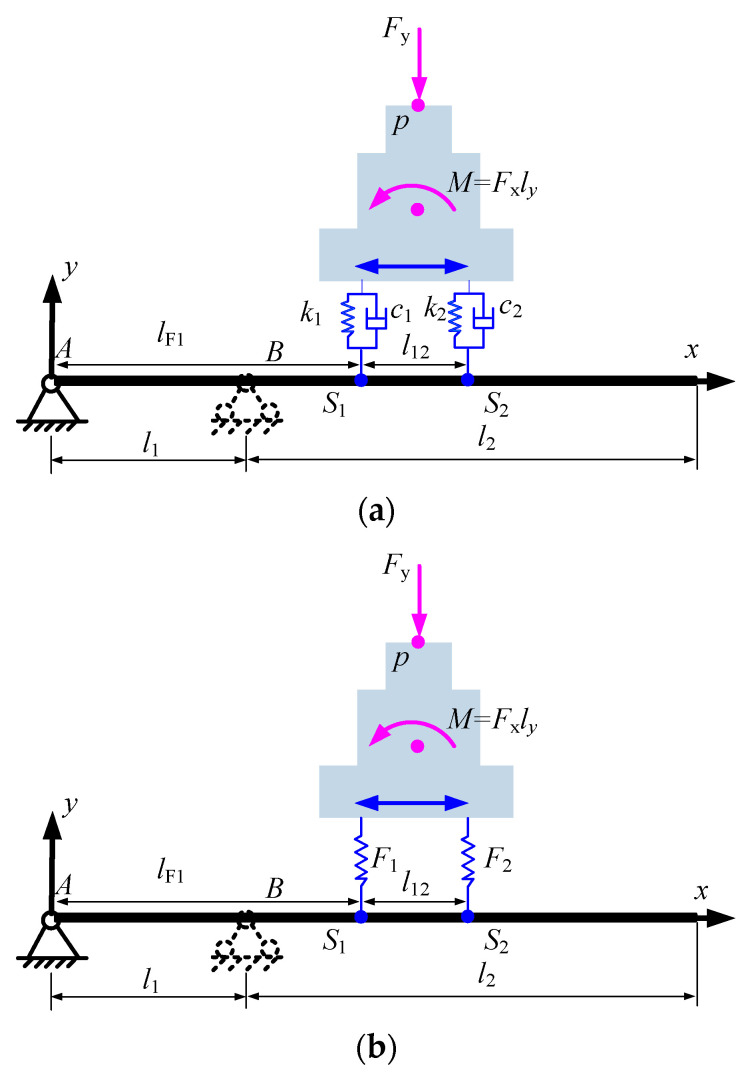
Schematic of rigid–flexible coupling system. (**a**) Simplification of loaded forces; (**b**) Model for statics analysis.

As Figure 2a shows, a reference coordinate system is established first, where the origin is located at the left end of the beam. The total length of the beam is denoted by *l*; the simply supported span is *l*_1_, which is variable; and the overhanging is *l*_2_, and *l* = *l*_1_ + *l*_2_. Two bodies are connected by the spring and damping with the stiffness *k*_1_, *k*_2_ and damping coefficients *c*_1_, *c*_2_, respectively. The distance from the left spring-damping unit to the left end of the beam is denoted by *l*_F1_, which varies along the *x*-axis direction, and the span between the two spring-damping units is a constant *l*_12_.

During the static analysis, the contact damping terms between the bodies are not taken into account, as shown in Figure 2b. According to the mechanics, the interaction forces *F*_1_ and *F*_2_ between the moving mass and elastic overhanging beam are

(1) $$\begin{array}{l} F_{1}=\frac{F_{y}}{2}+\frac{F_{x}l_{y}}{l_{12}}\\ F_{2}=\frac{F_{y}}{2}-\frac{F_{x}l_{y}}{l_{12}} \end{array}$$

**Figure 3:**
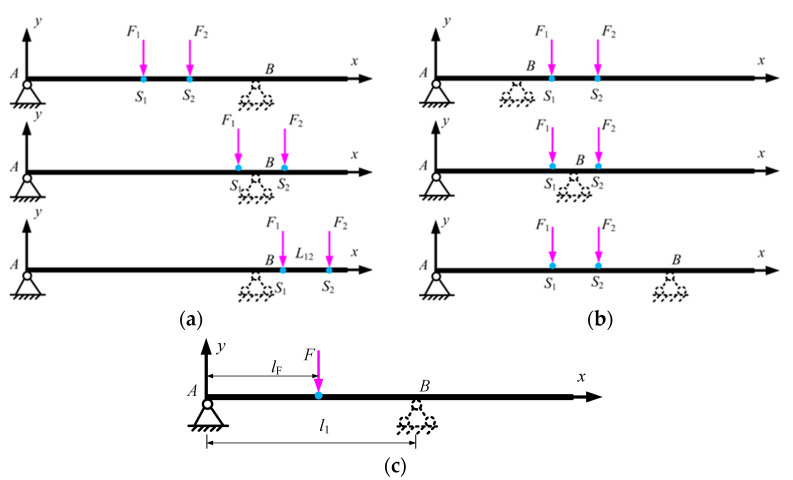
Equivalent model for static analysis. (**a**) The case that loads moving and support span fixed; (**b**) The case that support span varying and load position fixed; (**c**) General case of overhanging beam with single force.

There are two situations here: one is the mass moving along the *x* direction while the support position is fixed as shown in Figure 3a, and the other supporting position varies as shown in Figure 3b. For clarification, the calculation of the deflection at the point only the single force *F* is applied is given first and the schematic is shown in Figure 3c.

For the constant cross-section beam, the differential equation of the bending deflection *w* is

(2) $${EIw}^{\prime }=\int Mdx+C$$

where *E* is the elastic modulus of the beam material, *I* is the moment of inertia, and *C* is the constant to be determined according to the boundary conditions.

According to the mechanics of the material, substituting the boundary conditions into Equation (2), the deflection where force *F* is applied to the position is obtained:

(3) $$w=\left\{\begin{array}{cc} -\frac{Fl_{F}^{2}{\left(l_{1}-l_{F}\right)}^{2}}{3EIl_{1}} & l_{F}\leq l_{1}\\ -\frac{Fl_{F}{\left(l_{1}-l_{F}\right)}^{2}}{3EI} & l_{1}<l_{F} \end{array}\right.F\;=\;F_{1}\;\mathrm{or}\;F_{2}$$

**Figure 4:**
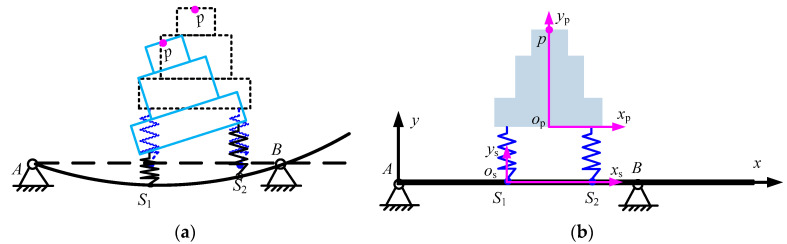
Deviation of reference point. (**a**) Schematic of deviation of point *p*; (**b**) Coordinates for describing deviation of point *p*.

In this case, there are two forces *F*_1_, *F*_2_ simultaneously applied; thus, the superposition method is employed to obtain the deflection at both points. To describe the deviation of point *p* referring to the original position, a homogenous transfer matrix is adopted here, as Figure 4 shows.

Two local coordinate systems are built: *o*_s_*x*_s_*y*_s_, whose origin *o*_s_ is fixed to point *S*_1_ and the *x*_s_-axis is always pointing toward *S*_2_, and *o*_p_*x*_p_*y*_p_, whose origin is set at the midpoint of two connecting springs and the *x*_p_-axis lies on the bottom of the rigid mass, shown in Figure 4b.

Taking *Axy* as the global coordinate system, the homogeneous coordinates of point *p* in the global aspect before loads are applied is denoted by [*p*_x0_,*p*_y0_,*p*_z0_,1]^T^, and

(4) $$\left[\begin{array}{c} p_{x0}\\ p_{x0}\\ p_{x0}\\ 1 \end{array}\right]=T_{Ao_{s}}T_{o_{s}o_{p}}\left[\begin{array}{c} 0\\ l_{y}\\ 0\\ 1 \end{array}\right]$$

where the transfer matrixes *T_Aos_*, *T_osop_* are

$$T_{Ao_{s}}=\left[\begin{array}{cccc} 1 & 0 & 0 & l_{F1}\\ 0 & 1 & 0 & 0\\ 0 & 0 & 1 & 0\\ 0 & 0 & 0 & 1 \end{array}\right],\;T_{o_{s}o_{p}}=\left[\begin{array}{cccc} 1 & 0 & 0 & l_{12}/2\\ 0 & 1 & 0 & 0\\ 0 & 0 & 1 & 0\\ 0 & 0 & 0 & 1 \end{array}\right].$$

As the deflections are small, the deviation matrixes under loads are

(5) $$\Delta T_{Ao_{s}}=\left[\begin{array}{cccc} 1 & -\gamma_{s} & 0 & 0\\ \gamma_{s} & 1 & 0 & w_{1}\\ 0 & 0 & 1 & 0\\ 0 & 0 & 0 & 1 \end{array}\right]$$

(6) $$\Delta T_{o_{s}o_{p}}=\left[\begin{array}{cccc} 1 & -\gamma_{p} & 0 & 0\\ \gamma_{p} & 1 & 0 & \delta_{y}\\ 0 & 0 & 1 & 0\\ 0 & 0 & 0 & 1 \end{array}\right]$$

where $\gamma_{s}\approx \frac{w_{2}-w_{1}}{l_{12}}$, $\gamma_{p}=\frac{F_{x}l_{y}}{k_{1}l_{12}^{2}}=\frac{F_{x}l_{y}}{k_{2}l_{12}^{2}}$, and $\delta_{y}=\frac{F_{y}}{k_{1}+k_{2}}$.

Then, the resultant coordinates of point *p* are obtained by

(7) $$\left[\begin{array}{c} p_{x}\\ p_{y}\\ p_{z}\\ 1 \end{array}\right]=T_{Ao_{s}}\Delta T_{Ao_{s}}T_{o_{s}o_{p}}\Delta T_{o_{s}o_{p}}\left[\begin{array}{c} 0\\ l_{y}\\ 0\\ 1 \end{array}\right]$$

The compound deviation amount is then calculated by

(8) $$\delta =\sqrt{{\left(p_{x}-p_{x0}\right)}^{2}+{\left(p_{y}-p_{y0}\right)}^{2}}$$

This is the evaluating indicator for the static analysis.

## 3. Frequency Response Functions

Reviewing Figure 1, the motion equations of the coupling system are given by

(9) $$m_{s}\ddot{y}+F_{d1}+F_{d2}=F_{y}$$

(10) $$J_{s}(\ddot{\theta }-\frac{{\ddot{y}}_{b1}-{\ddot{y}}_{b2}}{l_{12}})+\frac{F_{d1}l_{12}}{2}-\frac{F_{d2}l_{12}}{2}=F_{x}\frac{l_{y}}{2}$$

(11) $$m\frac{\partial^{2}y_{b}}{\partial t^{2}}+EI\frac{\partial^{4}y_{b}}{\partial x^{4}}=F_{d1}+F_{d2}$$

where *m*_s_, *J*_s_ represent the mass and moment of inertia of the moving component, and m is the mass per unit length of the beam. The variables *y*, *y*_b_ represent the vibration displacements of the lumped mass and beam along the *y*-axis, respectively. And *θ* is the angular displacement of the mass. The interaction forces *F*_d1_, *F*_d2_ between two bodies are given by

(12) $$F_{d1}=c_{1}(\dot{y}-{\dot{y}}_{b1}+\frac{l_{12}}{2}\dot{\theta })+k_{1}(y-y_{b1}+\frac{l_{12}}{2}\theta )$$

(13) $$F_{d2}=c_{2}(\dot{y}-{\dot{y}}_{b2}-\frac{l_{12}}{2}\dot{\theta })+k_{2}(y-y_{b2}-\frac{l_{12}}{2}\theta )$$

### 3.1. Vibration Mode of the Overhanging Beam

The free vibration equation of Equation (11) is written by

(14) $$m\frac{\partial^{2}y_{b}}{\partial t^{2}}+EI\frac{\partial^{4}y_{b}}{\partial x^{4}}=0$$

By means of the separation of variables and mode superposition, the solution of Equation (14) takes the following form:

(15) $$y_{b}(x,t)=\sum_{r=1}^{\infty }Y_{r}(x)q_{r}(t)$$

where *Y_r_*(*x*) is the *r*th vibration shape function, and *q*_r_(*t*) is the *r*th-order response in the time domain. Correspondingly, the response at the force-applied points is expressed by

(16) $$y_{b}(x_{1},t)=\sum_{r=1}^{\infty }Y_{r}(x_{1})q_{r}(t)=y_{b1}(t)$$

(17) $$y_{b}(x_{2},t)=\sum_{r=1}^{\infty }Y_{r}(x_{2})q_{r}(t)=y_{b2}(t)$$

**Figure 5:**
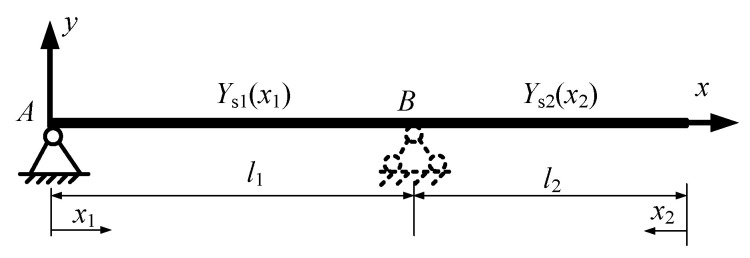
An overhanging beam.

To derive the mode function, the beam is divided into two segments, as shown in Figure 5.

The functions take the following forms:

(18) $$Y_{s1}(x_{1})=C_{1}S(\lambda x_{1})+C_{2}T(\lambda x_{1})+C_{3}U(\lambda x_{1})+C_{4}V(\lambda x_{1})$$

(19) $$Y_{s2}(x_{2})={C_{1}}^{\prime }S(\lambda x_{2})+{C_{2}}^{\prime }T(\lambda x_{2})+{C_{3}}^{\prime }U(\lambda x_{2})+{C_{4}}^{\prime }V(\lambda x_{2})$$

(20) $$\left\{\begin{array}{c} S(\lambda x)=\frac{1}{2}(ch\lambda x+\cos\lambda x)\\ T(\lambda x)=\frac{1}{2}(sh\lambda x+\sin\lambda x)\\ U(\lambda x)=\frac{1}{2}(ch\lambda x-\cos\lambda x)\\ V(\lambda x)=\frac{1}{2}(sh\lambda x-\sin\lambda x) \end{array}\right.,\;x\;=\;x_{1}\;\mathrm{or}\;x_{2}$$

where *λ* is an auxiliary variable $\lambda^{4}=\frac{\omega_{n}^{2}m}{EI}$, and *ω_n_* is the natural frequency.

The left end of the first segment is simply supported, and the boundary conditions are as follows:

(21) $$x_{1}=0,Y_{s1}(0)={Y_{s1}}^{\prime \prime }(0)=0$$

The right end of the second segment is free, and the boundary conditions are as follows:

(22) $$x_{2}=0,{Y_{s2}}^{\prime \prime }(0)={Y_{s2}}^{\prime \prime \prime }(0)=0$$

Moreover, the boundary conditions at the connection between the two segments are as follows:

(23) $$Y_{s1}(l_{1})=Y_{s2}(l_{2})=0,{Y_{s1}}^{\prime }(l_{1})={Y_{s2}}^{\prime }(l_{2}),{Y_{s1}}^{\prime \prime }(l_{1})={Y_{s2}}^{\prime \prime }(l_{2})$$

Substituting the above boundary conditions into Equations (18) and (19), the natural frequency equation is obtained,

(24) $$\frac{ch\lambda l_{1}\sin\lambda l_{1}-sh\lambda l_{1}\cos\lambda l_{1}}{2sh\lambda l_{1}\sin\lambda l_{1}}=\frac{ch\lambda l_{2}\cos\lambda l_{2}+1}{ch\lambda l_{2}\sin\lambda l_{2}-sh\lambda l_{2}\cos\lambda l_{2}}$$

where $\omega_{nr}=\lambda_{r}^{2}\sqrt{\frac{EI}{m}}$.

And the shape functions are

(25) $$Y_{s1}(x_{1})=D\left(\sin\lambda x_{1}-\frac{\sin\lambda l_{1}}{sh\lambda l_{1}}sh\lambda x_{1}\right)$$

(26) $$Y_{s2}(x_{2})=D\left(\begin{array}{l} ch\lambda x_{2}+\cos\lambda x_{2}-\\ \frac{ch\lambda l_{2}+\cos\lambda l_{2}}{sh\lambda l_{2}+\sin\lambda l_{2}}(sh\lambda x_{2}+\sin\lambda x_{2}) \end{array}\right)$$

(27) $$D=\sqrt{\frac{4\lambda }{m\left(den1+den2+den3+den4+den5\right)}}$$

where

$$\begin{array}{l} den1=2\lambda l_{1}{\left(1-\frac{\sin\lambda l_{1}}{sh\lambda l_{1}}\right)}^{2}\\ den2=2\sin\lambda l_{1}\left(sh\lambda l_{1}\cos\lambda l_{1}-ch\lambda l_{1}\sin\lambda l_{1}\right)/sh\lambda l_{1}\\ den3=4\lambda l_{2}+(1+{C_{a}}^{2})sh2\lambda l_{2}\\ den4=(1-{C_{a}}^{2})\sin2\lambda l_{2}-2C_{a}(ch2\lambda l_{2}-\cos2\lambda l_{2})+4(1+{C_{a}}^{2})ch\lambda l_{2}\sin\lambda l_{2}\\ den5=4(1-{C_{a}}^{2})sh\lambda l_{2}\cos\lambda l_{2}-8Csh\lambda l_{2}\sin\lambda l_{2}\\ C_{a}=\frac{ch\lambda l_{2}+\cos\lambda l_{2}}{sh\lambda l_{2}+\sin\lambda l_{2}} \end{array}$$

### 3.2. Frequency Response Function

Substituting Equation (15) and its derivatives into Equation (11) yields

(28) $${\ddot{q}}_{r}(t)+\omega_{nr}^{2}q_{r}(t)=Q_{r}(t)\;r\;=\;1,\;2,\;\ldots$$

As above, the individual equations are independent of each other and take the same form with the motion equation of the single degree of freedom without damping; thus, the solution *q_r_*(*t*) takes the following form:

(29) $$q_{r}(t)=\frac{1}{\omega_{nr}}\int_{0}^{t}Q_{r}(\tau )\sin\omega_{nr}(t-\tau )d\tau$$

For the concentrated load, the *r*th-order generalized force *Q_r_*(*t*) takes the following form:

(30) $$Q_{r}(t)=F_{d1}(t)Y_{r}(x_{1})+F_{d2}(t)Y_{r}(x_{2})$$

Consequently,

(31) $$q_{r}(t)=\frac{Y_{r}(x_{1})}{\omega_{nr}}\int_{0}^{t}F_{d1}(\tau )\sin\omega_{nr}(t-\tau )d\tau +\frac{Y_{r}(x_{2})}{\omega_{nr}}\int_{0}^{t}F_{d2}(\tau )\sin\omega_{nr}(t-\tau )d\tau$$

Performing the Laplace transform on Equation (15),

(32) $$L(y_{b})=\sum_{r=1}^{\infty }Y_{r}(x)L(q_{r})$$

By introducing the convolution transform, *L*(*q_r_*) is obtained:

(33) $$L(q_{r})=\frac{Y_{r}(x_{1})}{\omega_{nr}^{2}+s^{2}}F_{d1}(s)+\frac{Y_{r}(x_{2})}{\omega_{nr}^{2}+s^{2}}F_{d2}(s)$$

Correspondingly, the Laplace transform of Equations (16) and (17) results in

(34) $$\left[\begin{array}{c} Y_{b1}(s)\\ Y_{b2}(s) \end{array}\right]=C\left[\begin{array}{c} F_{d1}(s)\\ F_{d2}(s) \end{array}\right]$$

where

$$C=\left[\begin{array}{cc} \sum_{r=1}^{\infty }\frac{Y_{r}^{2}(x_{1})}{\omega_{nr}^{2}+s^{2}} & \sum_{r=1}^{\infty }\frac{Y_{r}(x_{1})Y_{r}(x_{2})}{\omega_{nr}^{2}+s^{2}}\\ \sum_{r=1}^{\infty }\frac{Y_{r}(x_{1})Y_{r}(x_{2})}{\omega_{nr}^{2}+s^{2}} & \sum_{r=1}^{\infty }\frac{Y_{r}^{2}(x_{2})}{\omega_{nr}^{2}+s^{2}} \end{array}\right]$$

Similarly, the Laplace transform of Equations (12) and (13) gives

(35) $$\left[\begin{array}{c} F_{d1}(s)\\ F_{d2}(s) \end{array}\right]=(cs+k)\left[\begin{array}{cc} 1 & l_{12}/2\\ 1 & -l_{12}/2 \end{array}\right]\left[\begin{array}{c} Y\\ \theta \end{array}\right]+(cs+k)\left[\begin{array}{cc} -1 & 0\\ 0 & -1 \end{array}\right]\left[\begin{array}{c} Y_{b1}\\ Y_{b2} \end{array}\right]$$

Substituting Equation (34) into Equation (35) yields

(36) $$\left[\begin{array}{c} F_{d1}(s)\\ F_{d2}(s) \end{array}\right]=K\left[\begin{array}{c} Y\\ \theta \end{array}\right]$$

where

(37) $$K=(cs+k){\left(E_{2\times 2}-(cs+k)\left[\begin{array}{cc} -1 & 0\\ 0 & -1 \end{array}\right]\times C\right)}^{-1}\left[\begin{array}{cc} 1 & l_{12}/2\\ 1 & -l_{12}/2 \end{array}\right]$$

Thus, the relationship between *Y_b_*_1_, *Y_b_*_2_ and *Y*, *θ* could be determined by

(38) $$\left[\begin{array}{c} Y_{b1}(s)\\ Y_{b2}(s) \end{array}\right]=C\left[\begin{array}{c} F_{d1}(s)\\ F_{d2}(s) \end{array}\right]=C\times K\left[\begin{array}{c} Y\\ \theta \end{array}\right]$$

The Laplace transforms of Equations (9) and (10) are written as

(39) $$M\left[\begin{array}{c} Y\\ \theta \end{array}\right]+C_{F}\left[\begin{array}{c} F_{d1}\\ F_{d2} \end{array}\right]+K_{yb}\left[\begin{array}{c} Y_{b1}\\ Y_{b2} \end{array}\right]=\left[\begin{array}{c} F_{y}\\ F_{x} \end{array}\right]$$

where

$$M=\left[\begin{array}{cc} m_{s}s^{2} & 0\\ 0 & \frac{2J_{s}s^{2}}{l_{y}} \end{array}\right],K_{yb}=\left[\begin{array}{cc} 0 & 0\\ -\frac{2J_{s}s^{2}}{l_{12}l_{y}} & \frac{2J_{s}s^{2}}{l_{12}l_{y}} \end{array}\right],C_{F}=\left[\begin{array}{cc} 1 & 1\\ \frac{l_{12}}{l_{y}} & -\frac{l_{12}}{l_{y}} \end{array}\right].$$

Combining Equations (36)–(39) yields

(40) $$\left(M+C_{F}\times K+K_{yb}\times C\times K\right)\left[\begin{array}{c} Y\\ \theta \end{array}\right]=\left[\begin{array}{c} F_{y}\\ F_{x} \end{array}\right]$$

Finally, the matrix ***H*** of the frequency response function is obtained by calculating the inverse matrix of the left hand side of the above equation and replacing *s* with *jω*,

(41) $$\left[\begin{array}{c} Y\\ \theta \end{array}\right]=H(j\omega )\left[\begin{array}{c} F_{y}\\ F_{x} \end{array}\right]=\left[\begin{array}{cc} H_{11} & H_{12}\\ H_{21} & H_{22} \end{array}\right]\left[\begin{array}{c} F_{y}\\ F_{x} \end{array}\right]$$

(42) $$H(j\omega )={\left(\left[\begin{array}{cc} -m_{s}\omega^{2} & 0\\ 0 & -\frac{2J_{s}\omega^{2}}{l_{y}} \end{array}\right]+\left[\begin{array}{cc} 1 & 1\\ \frac{l_{12}}{l_{y}} & -\frac{l_{12}}{l_{y}} \end{array}\right]\times K+\left[\begin{array}{cc} 0 & 0\\ \frac{2J_{s}\omega^{2}}{l_{12}l_{y}} & -\frac{2J_{s}\omega^{2}}{l_{12}l_{y}} \end{array}\right]C\times K\right)}^{-1}$$

where *ω* is the exciting frequency.

## 4. Simulation and Discussion

In this section, the influences of the support position of a rigid mass–flexible beam on both the static and frequency response characteristics of a given example are studied. The parameters used in the simulation are given in Table 1.

### 4.1. Static Deflection Analysis

**Figure 6:**
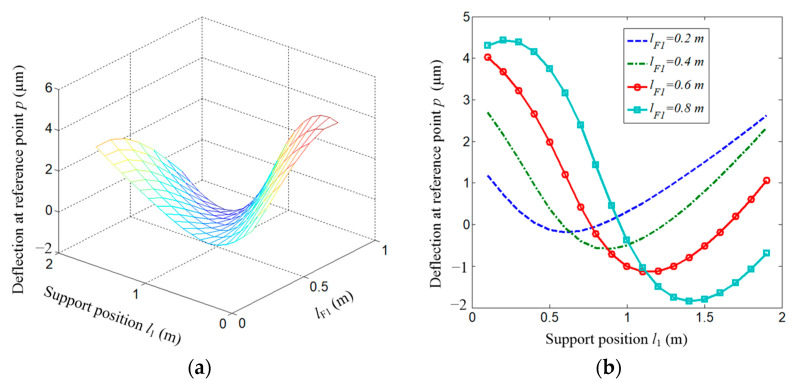
Deflection along *x*-axis direction. (**a**) Distribution of deflection along *x*-axis at point *p*; (**b**) Several extracted cases from (**a**).

**Figure 7:**
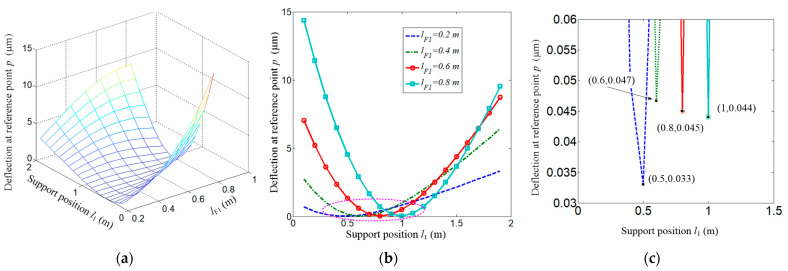
Deflection along *y*-axis direction. (**a**) Distribution of deflection along *y*-axis at point *p*; (**b**) Several extracted cases from (**a**); (**c**) partial enlargement view of (**b**).

**Figure 8:**
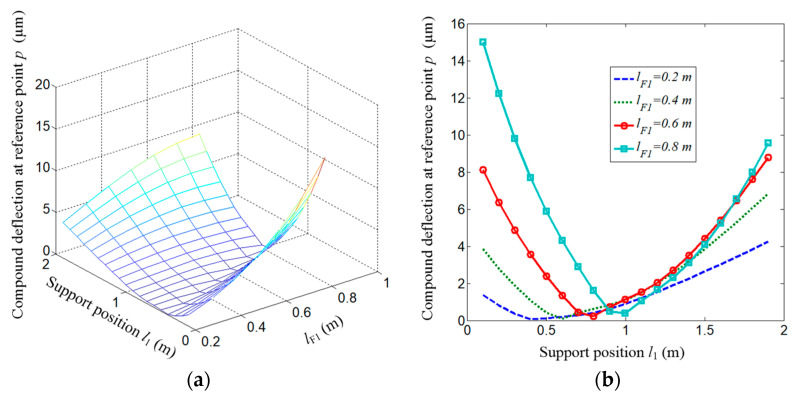
Compound deflection at reference point. (**a**) Compound deflection at point *p*; (**b**) Several extracted cases from (**a**).

In the simulation, the forces *F*_x_ = 200 N and *F*_y_ = 1000 N are applied. The individual deflection along the *x*, *y*-axis and the compound deflection of reference point *p* are shown in Figure 6, Figure 7 and Figure 8.

Figure 6a shows the variation in the deflection along the *x*-axis with a different supporting span *l*_1_ and displacement over the travel of the moving mass. As the *x*-axis is longitudinal, the deflection at the reference point may be positive or negative. To see clearly, Figure 6b gives the results under several displacements of the mass. As the figure shows, the changing trends are similar with different force-applied positions *l*_F1_ = 0.2, 0.4, 0.6, and 0.8 m.

Taking *l*_F1_ = 0.4 m as an example, the deflection is along the positive direction of the *x*-axis when the supporting span is less than 0.58 m and decreases with the span *l*_1_ increasing. At two special supporting positions, *l*_1_ = 0.58 and 1.24 m, the deflections are zero and negative. When the span is longer than *l*_1_ = 1.24, the deflection increases with *l*_1_.

Similarly, the deflection along the *y*-axis is shown in Figure 7. The deflection is always along the minus direction of the *y*-axis and the absolute value is given. Figure 7a shows the deflection distribution. Figure 7b gives the deflection curves with different force-applied positions.

It could be seen from the figure that once the force-applied position is fixed, there definitely exists an inflection point where the deflection is at a minimum. Specifically, as shown in Figure 7c, the optimal supporting spans are 0.5, 0.6, 0.8, and 1 m when the force-applied position is *l*_F1_ = 0.2, 0.4, 0.6, and 0.8 m, respectively.

The compound deflection defined by Equation (8) is shown in Figure 8. As the deflection along the y direction is larger than those along the *x*-axis, consequently, the inflection point is very close to those shown in Figure 7b.

In real practice, the relative deflection over the total travel of the moving rigid mass may be more interesting. Thus, the relative deflection *δ*_re_ is defined as

*δ*_re_ = *w*_max_ − *w*_min_ (43)

where *w*_max_, *w*_min_ represent the maximum and minimum deflection over the total travel under a given supporting span, respectively.

**Figure 9:**
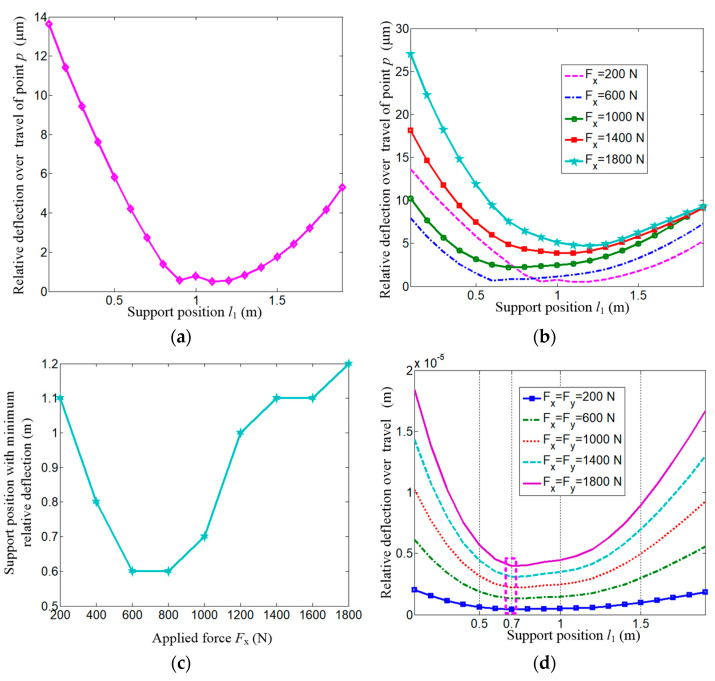
Relative deflection under different loads.

Figure 9a shows the relative deflection *δ* over the total travel of reference point *p*. The value does not change monotonically but reaches its minimum at a span of *l*_1_ = 1.16 m. It should be noted that the result comes from *F*_x_ = 200 N and *F*_y_ = 1000 N. Whether there are influences of applied forces on the relative value, Figure 9b gives the results where *F*_y_ = 1000 N and *F*_x_ varies uniformly from 200 N to 1800 N. Obviously, the trends are similar, but the inflection points differ under different loading conditions. Figure 9c shows the optimal supporting span with the minimum relative deflection versus the load *F*_x_. Moreover, the case *F*_x_ = *F*_y_ is also studied, which is shown in Figure 9d. It could be seen from the figure that whatever the specific value of the force load is, the optimal supporting span is *l*_1_ = 0.7 m.

**Figure 10:**
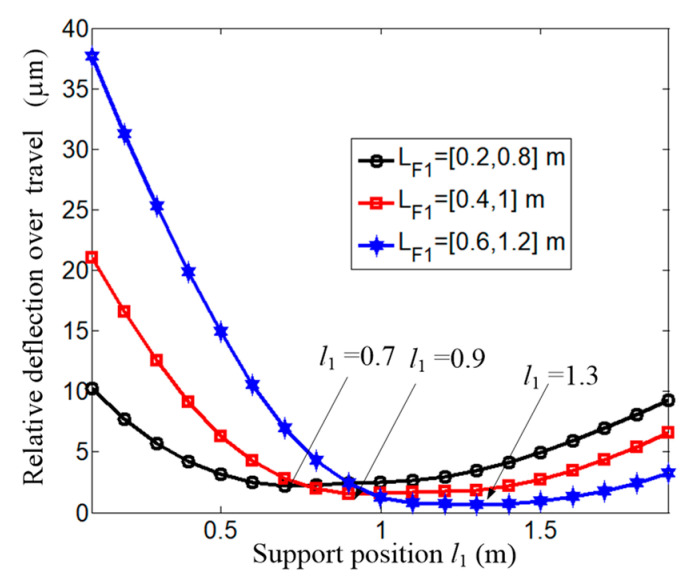
Relative deflection under different travel values.

Besides the applied load, whether the travel affects the optimal supporting position or not is checked further. The travels selected are [0.2, 0.8], [0.4, 1], and [0.6, 1.2] m, respectively. The applied forces are *F*_x_ = *F*_y_ = 1000 N. The results are given in Figure 10. The supporting span with the minimum relative deflection for the three travels is *l*_1_ = 0.7, 0.9, and 1.3 m, respectively. That means that the optimal supporting span is related to the starting position of the total travel.

Based on the above analysis, here, a conclusion can be drawn: for the given specific travel and applied forces, there always exists a supporting span minimizing the relative deflection over the total travel. And the optimal value depends on the relationship between the loads in two directions and the starting position of the travel.

### 4.2. Frequency Response Analysis

The static analysis is executed in the above section, and this part will show the influence of the supporting span on the dynamic characteristics of the reference point by means of the frequency responses. The exciting frequency is up to 1500 Hz and *l*_F1_ = 0.8 m.

**Figure 11:**
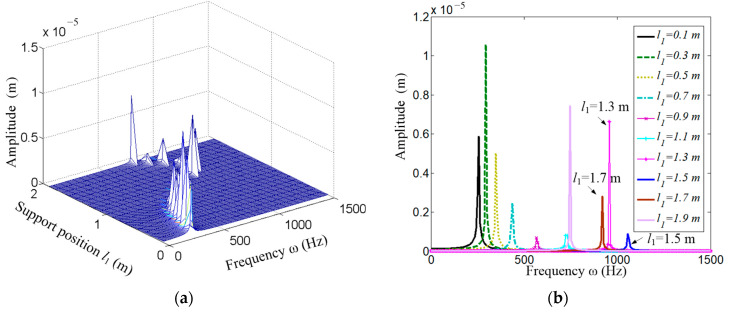

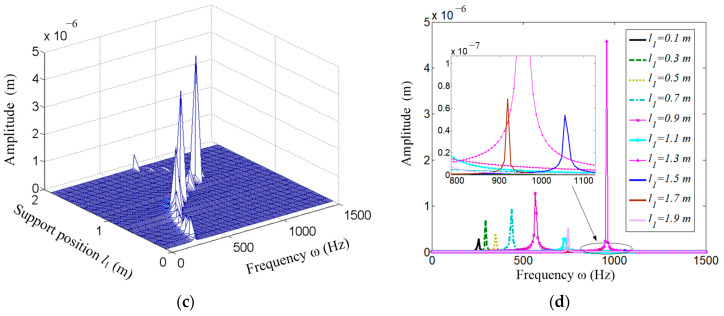
Frequency response function *H*_11_ and *H*_12_. (**a**) Distribution of amplitude of function *H*_11_. (**b**) Several extracted cases from (**a**); (**c**) Distribution of amplitude of function *H*_12_; (**d**) Several extracted cases from (**c**).

Recalling Equation (35), *H*_11_, *H*_12_ represent the response of the vibration displacement y in the frequency domain under excitations from *F*_y_, *F*_x_, respectively. As the first-order frequency is more significant than the others, only the first is extracted in the simulation, and the results are shown in Figure 11a–d. Figure 11a,c give the variation in the amplitude thorough the supporting span [0.1, 1.9] m under a series of exciting frequencies. Figure 11b,d show this at several discrete spans. It is not difficult to find that the first-order resonant frequency increases first and then decreases with the increasing of the supporting span. Taking Figure 11b as an example, the frequency reaches its maximum when the span *l*_1_ = 1.5 m, which is higher than those both at *l*_1_ = 1.3m and *l*_1_ = 1.7 m.

A similar situation could be found in the cross-frequency response function *H*_12_, which is not described further.

**Figure 12:**
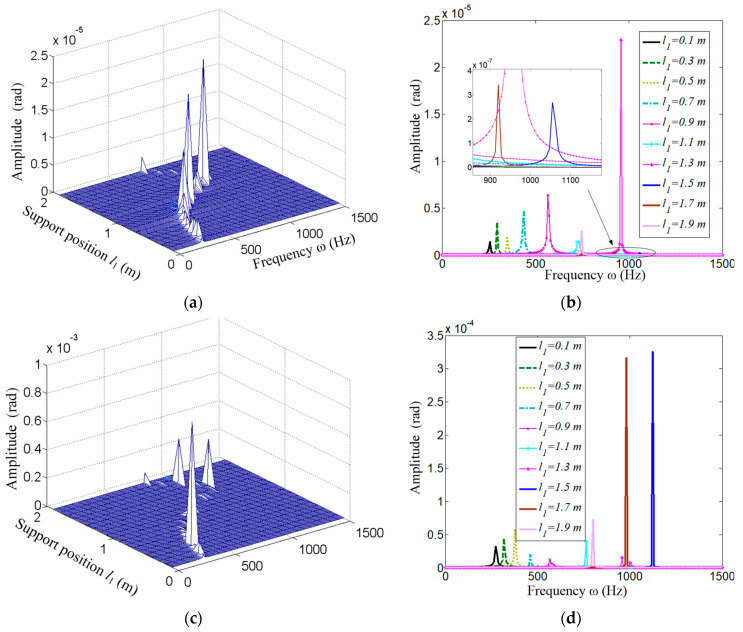
Frequency response function *H*_21_ and *H*_22_. (**a**) Distribution of amplitude of function *H*_21_; (**b**) Several extracted cases from (**a**); (**c**) Distribution of amplitude of function *H*_22_; (**d**) Several extracted cases from (**c**).

The frequency responses of the angular displacement are shown in Figure 12. Figure 12a,b are the cross function *H*_12_ and Figure 12c,d shows *H*_22_.

**Figure 13:**
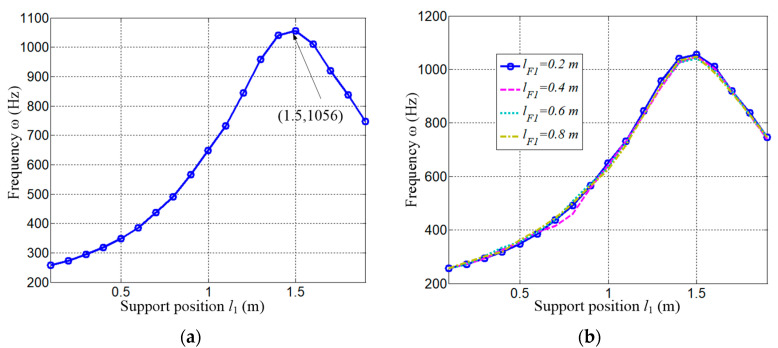
The first resonant frequency varying with the supporting position. (**a**) The frequency with *l*_F1_ = 0.2 m; (**b**) The frequency with *l*_F1_ = 0.2 m, 0.4 m, 0.6 m, 0.8 m.

As the figures show, the maximum resonant frequency is obtained when the span *l*_1_ = 1.5 m, and the same with *H*_11_ and *H*_12_. To further clarify the effect on the resonant frequency, the relationship between the supporting span and the frequency is given in Figure 13.

Figure 13a indicates that the increase in the supporting span could effectively improve the first resonant frequency of the rigid–flexible coupling system up to *l*_1_ = 1.5 m, and the maximum value is about 1056 Hz. However, a too long span will have a negative influence, as the frequency decreases when *l*_1_ is greater than 1.5 m. The effect of the force-applied position is checked and shown in Figure 13b, which implies that there is a little variation in the frequency. This is because the rigid mass is 20 kg, which is much less than the beam mass of 156 kg, and the mass distribution of the coupled system changes little while the rigid body is moving.

**Figure 14:**
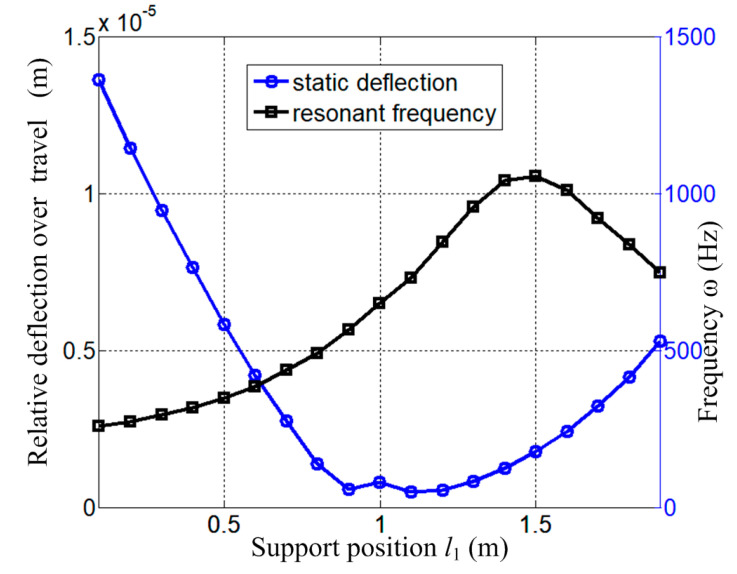
Effect of supporting position on both static deflection and resonant frequency.

To further reveal the influence of the supporting span on both the statics and dynamics of the coupled system, the relative static deflection over the total travel and the resonant frequency are shown in Figure 14.

The simulation conditions are *F*_x_ = 200 N, *F*_y_ = 1000 N, and *l*_F1_ = 0.8m. As the figure shows, the optimal supporting spans for the individual static and dynamic conditions are usually not the same: *l*_1_ = 1.1m for the minimum relative static deflection and *l*_1_ = 1.5 m for the maximum resonant frequency. In practice, a specific supporting span could be determined by combining both the static and dynamic constraints. For example, the relative deflection is specified to be no more than 5 μm, the load exciting frequency is lower than 500 Hz, and then the span of *l*_1_ = 0.85~1.85 m could be chosen as the candidates.

## 5. Conclusions

This work conducted a study about the influence of the height difference of guideways on the performance of a horizontal machining center. The following conclusions could be drawn:

(1) Given working conditions like stroke and applied forces, there exists an optimal height difference that minimizes the relative static deflection over the stroke;
(2) The relationship between applied forces along two orthogonal directions affects the specific optimal value of the height difference corresponding to the minimum relative deflection. Moreover, the same stroke but different launching positions also alters the optimal height difference.
(3) The first-order resonant frequency does not change monotonically with increasing height difference, and there always exists an optimal position that results in the highest resonant frequency.

As the optimal value for the statics is not the same as that of the dynamics, in practice, a reasonable height difference could be determined simultaneously considering both the static and dynamic constraints.

It is known that thermal deformation and guideway wear will affect the performance of machine tools; in the future, optimization considering these factors should be further investigated.

## Figures and Tables

**Table 1:** Simulation condition.

Total length *l* (m)	2	Force arm *l*_y_ (m)	0.4	Density (kg/m^3^)	7800
Range of *l*_1_ (m)	0.1~1.9	Transverse section of beam *b* × *h* mm	100 × 200	Contact stiffness (N/m)	2 × 10^9^
*l*_F1_ (m)	0.2~0.8	Mass of moving part (kg)	20	Contact damping (N.s/m)	2 × 10^6^
*l*_12_ (m)	0.4	Moment of inertia (kg.m^2^)	0.003		

## Data Availability

The original contributions presented in this study are included in the article.
